# Navigating Equitable Access to Cancer and Mental Health Services During Pandemics: Stakeholder Perspectives on COVID-19 Challenges and Community-Based Solutions for Immigrants and Refugees—Proceedings from Think Tank Sessions

**DOI:** 10.3390/healthcare13050564

**Published:** 2025-03-05

**Authors:** Mandana Vahabi, Kimberly Devotta, Cliff Ledwos, Josephine P. Wong, Miya Narushima, Jennifer Rayner, Roula Hawa, Kenneth Fung, Geetanjali D. Datta, Axelle Janczur, Cynthia Damba, Aisha Lofters

**Affiliations:** 1 Lawrence Bloomberg Faculty of Nursing, University of Toronto, Toronto, ON M5T 1P8, Canada; 2 Li Ka Shing Knowledge Institute, Unity Health Toronto, St. Michael’s Hospital, Toronto, ON M5B 1W8, Canada; 3 ICES, Toronto, ON M4N 3M5, Canada; 4 MAP Centre for Urban Health Solutions, Li Ka Shing Knowledge Institute, St. Michael’s Hospital, Toronto, ON M5B 1W8, Canada; 5 Daphne Cockwell School of Nursing, Toronto Metropolitan University, Toronto, ON M5B 2K3, Canada; jph.wong@torontomu.ca; 6 Peter Gilgan Centre for Women’s Cancers, Women’s College Hospital, Toronto, ON M5S 1B2, Canada; kimberly.devotta@wchospital.ca (K.D.); aisha.lofters@wchospital.ca (A.L.); 7 Access Alliance Multicultural Health and Community Services, Toronto, ON M1L 1A8, Canada; cledwos@accessalliance.ca (C.L.); ajanczur@accessalliance.ca (A.J.); 8 Dalla Lana School of Public Health, University of Toronto, Toronto, ON M5T 3M7, Canada; 9 Department of Health Sciences, Brock University, St. Catharines, ON L2S 3A1, Canada; mnarushima@brocku.ca; 10 Department of Family and Community Medicine, University of Toronto, Toronto, ON M5S 1A1, Canada; jennifer.rayner@allianceon.org; 11 Alliance for Healthier Communities, North York, ON M6A 3B6, Canada; 12 Family Studies & Human Development, Faculty of Health Sciences, Western University, London, ON N6A 3K7, Canada; roula.hawa@uwo.ca; 13 Asian Initiative in Mental Health, Global Mental Health, Department of Psychiatry, University of Toronto, Toronto, ON M5G 1X5, Canada; ken.fung@uhn.ca; 14 Toronto Western Hospital, Toronto, ON M5T 2S8, Canada; 15 Cancer Research Center for Health Equity, Cedars-Sinai Medical Center, Los Angeles, CA 90048, USA; geetanjali.datta@cshs.org; 16 Department of Medicine, Cedars-Sinai Medical Center, Los Angeles, CA 90048, USA; 17 Ontario Health Toronto, Toronto, ON M5G 2L3, Canada; cynthia.damba@ontariohealth.ca

**Keywords:** immigrant, refugees, COVID-19, Think Tank, community-based solutions, Ontario, health inequities, cancer, mental health and addiction disorders

## Abstract

**Background:** Increasing evidence shows that the COVID-19 pandemic has disproportionately impacted certain populations, particularly those facing structural marginalization, such as immigrants and refugees. Additionally, research highlights that structurally marginalized populations living with chronic conditions, such as cancer and/or mental health and addiction (MH&A) disorders, are more vulnerable to the adverse effects of COVID-19. These individuals face higher susceptibility to infection and worse health outcomes, including increased rates of hospitalization, severe illness, and death. To better understand the challenges faced by people living at the intersection of social and clinical disadvantages, we organized a series of Think Tank sessions to engage stakeholders in exploring barriers and identifying community-based solutions for immigrants and refugees living with cancer and/or MH&A disorders during the current and future pandemics. **Objectives:** Our main objectives were to gauge how earlier findings resonated with stakeholders, to identify any gaps in the work, and to co-develop actionable solutions to safeguard health and well-being during COVID-19 and future crises. **Methods:** Two virtual Think Tank sessions were held in September 2023 as integrative knowledge exchange forums. The Cancer Think Tank was attended by 40 participants, while the MH&A disorders Think Tank included 41 participants. Each group comprised immigrants and refugees living with or affected by cancer (in the Cancer Think Tank) or MH&A disorders (in the MH&A disorders Think Tank), alongside service providers, policymakers, and researchers from Ontario. This paper presents the key discussions and outcomes of these sessions. **Results:** Participants identified and prioritized actionable strategies during the Think Tank sessions. In the Cancer Think Tank, participants emphasized the importance of leveraging foreign-trained healthcare providers to address workforce shortages, creating clinical health ambassadors to bridge gaps in care, and connecting immigrants with healthcare providers immediately upon their arrival in Canada. In the MH&A disorders Think Tank, participants highlighted the need to remove silos by fostering intersectoral collaboration, empowering communities and building capacity to support mental health, and moving away from one-size-fits-all approaches to develop tailored interventions that better address diverse needs. **Conclusions:** The Think Tank sessions enhanced our understanding of how the COVID-19 pandemic has impacted immigrants and refugees living with cancer and/or MH&A disorders. The insights gained informed a series of actionable recommendations to address the unique needs of these populations during the current pandemic and in future public health crises.

## 1. Introduction

The COVID-19 pandemic has had far-reaching effects across the globe. As jurisdictions continue to assess the impacts of this public health crisis on various populations, it has become increasingly evident that certain groups have faced greater vulnerabilities than others. In Canada, these impacts have disproportionately affected structurally marginalized communities. For instance, immigrants, refugees and racialized Canadians have experienced higher rates of COVID-19 cases, hospitalizations and deaths compared to non-immigrants and white Canadians [1,2,3,4,5].

In Ontario—Canada’s most populous province—immigrants and refugees at one point accounted for more than half of confirmed COVID-19 cases, despite comprising only about a quarter of the population [6]. Evidence suggests that immigrants and refugees are particularly vulnerable to COVID-19 and its adverse effects due to their structurally marginalized positions. Factors such as low income, precarious or underemployment, immigration status, and daily experiences of discrimination and bias contribute to this heightened vulnerability [4,5,7,8,9,10,11,12,13].

Existing research also shows that preexisting health conditions, such as cancer, as well as mental health and addiction (MH&A) disorders, increase susceptibility to COVID-19, placing individuals at a clinical disadvantage [14,15,16]. However, limited information exists on how COVID-19 has affected those living at the intersection of social and clinical disadvantages.

To address this gap, we conducted population-based retrospective cohort studies using linked Ontario administrative health databases in Phase 1 of this work [17,18]. Our analysis examined the impact of COVID-19 on immigrants and refugees (hereafter referred to as “immigrants”) living with cancer or MH&A disorders, making it the first study in Ontario to carry this out. Our findings revealed that immigrants with cancer or MH&A were more likely than non-immigrants without these conditions to reside in socially deprived neighborhoods and experience poverty. Additionally, immigrants with cancer or MH&A experienced worse COVID-19 outcomes compared to both non-immigrants with similar conditions and those without these conditions. Specifically, immigrants with active cancer were three times more likely to be hospitalized and admitted to intensive care units (ICU), and four times more likely to die from COVID-19 compared to non-immigrants without cancer [18]. Immigrants with MH&A were 52% more likely to be diagnosed with COVID-19, 65% more likely to die, and more than twice as likely to be hospitalized and admitted to ICU compared to non-immigrants without MH&A [17].

For further details, please refer to Silent Voices of Immigrants and Refugees Battling with Mental Health and Addiction During COVID-19: A Follow-Up Population-Based Cohort Retrospective Study in Ontario, Canada, Journal of Environmental Science and Public Health, 8(3): 150–167, https://doi.org/10.26502/jesph.96120213 [17], and The Impact of COVID-19 on Immigrants and Refugees Living with Cancer: A Population-Based Cohort Study in Ontario, Canada, Journal of Environmental Science and Public Health, 8(2): 116–132, https://doi.org/10.26502/jesph.96120210 [18].

The next phase of our work involved hosting two half-day think tank sessions to present our findings on COVID-19 outcomes and to discuss systemic barriers to care access with stakeholders. The primary goal was to foster knowledge exchange and critical dialog on strategic actions to address health disparities through policy and programming. Our aim was to develop inclusive, effective responses to reduce health disparities and promote equitable access to cancer and MH&A services during pandemics and future crises. The main objectives of these sessions were as follows:

(i) To gauge how the findings from Phase 1 resonated with stakeholders;
(ii) To identify any gaps in the Phase 1 findings;
(iii) To collaboratively develop actionable solutions to protect health and well-being during COVID-19 and future crises.

## 2. Description of Think Tank and Methods

In September 2023, two virtual Think Tank sessions were held to explore the compounded impacts of COVID-19 on immigrants living with cancer (first session: Wednesday, 27 September 2023, 9:00 AM–12:30 PM) and mental health and addiction (MH&A) disorders (second session: Thursday, 28 September 2023, 9:00 AM–12:30 PM). We chose the Think Tank approach because it serves as a structured medium for knowledge transfer and exchange, allowing for more strategic and solution-oriented discussions. Think Tanks have demonstrated success in being a way to bring stakeholders together to process and apply policy advice based off of research findings [19,20]. These sessions were designed as integrative knowledge exchange forums, bringing together individuals affected by cancer or MH&A disorders, service providers, policymakers, and researchers from Ontario. The heterogeneous patient groups were selected based on their vulnerability to the compounded effects of the COVID-19 pandemic, specifically immigrants and refugees living with cancer or mental health and addiction disorders. Our community partners facilitated recruitment by promoting the Think Tank session to their clients who were affected by these conditions. Given the trust and safety concerns that many immigrants and refugees may have, it was crucial to have someone they were comfortable with introduce them to the sessions. This careful approach helped foster engagement in the Think Tank discussions. To address potential language barriers, informal caregivers were also invited through our collaborating community-based agencies, as they often served as the primary contact for their clients. Additionally, we compiled a list of service providers and policymakers in Ontario involved in planning and delivering cancer or MH&A services, including organizations such as Canadian Mental Health Association, Ontario Health-Cancer Care Ontario. The inclusion criteria encompassed immigrant and refugee adults (aged 21 or older) who were either living with or affected by cancer or MH&A disorders, as well as individuals directly or indirectly involved in the development, implementation, delivery, or evaluation of related services.

Prior to the start of the think tank all participants received a copy of study findings via email. Each session was led by a team of external professional facilitators. Activities were conducted in English, with interpretation services available upon request. Participants were compensated with a $90 honorarium for completing the online questionnaire and participating in the Think Tank, plus an additional $45 upon request for internet expenses if they had unstable internet access.

Each Think Tank session consisted of three main components, summarized in Table 1.

### 2.1. Introduction and Panel Discussion to Understand How Phase 1 Findings Resonate with Stakeholders (60 min)

The session began with an introduction of panelists, followed by a presentation from the research team on existing evidence and data regarding the social and structural determinants of health inequities experienced by immigrants living with cancer and MH&A disorders during the COVID-19 pandemic. This was followed by a moderated panel discussion, including at least one patient/caregiver representative, a healthcare provider, a policy/decision-maker and two members of the research team.

### 2.2. Small Group Discussions to Identify Data Gaps in the Phase 1 Findings (60 min)

Participants were then divided into small breakout groups to discuss their reactions to the presented data and share ideas for action or change.

### 2.3. Plenary Session to Collaboratively Develop Actionable Solutions (30–60 min)

Participants reconvened in a plenary session to share key points from their small group discussions. Small group facilitators compiled a list of priority action items, which the group reviewed and expanded upon. Participants then voted on their top three priority actions and the three actions they felt would be easiest to implement.

Both Think Tank sessions were recorded via Zoom (https://www.zoom.com/) and transcribed verbatim by our research assistant, along with notes taken by the small group facilitators during the discussions. The Think Tank moderator then applied thematic analysis to identify key themes among the action and change ideas that emerged.

This study received Research Ethics Board approval from Toronto Metropolitan University (#2023-100) and Women’s College Hospital (#2023-0027-E).

In the following section, we present the outcomes of each of the three components of the Think Tank activities and how they informed recommendations for responding to pandemics and future crises.

## 3. Findings from the Think Tank

The Think Tanks on cancer and MH&A disorders were attended by 40 and 41 participants, respectively. These groups comprised immigrants and refugees living with or affected by cancer (in the cancer Think Tank) or MH&A disorders (in the MH&A disorders Think Tank), along with service providers, and policy/decision-makers. A more detailed breakdown of the groups can be found in Table 2.

### 3.1. Cancer and MH&A Disorders Panels

During the Cancer panel discussion, two research team members who presented the study findings (M.V. and A.L.), a patient/caregiver, and a healthcare provider/policymaker engaged in a discussion about the research outcomes, described above like significantly higher rates of hospitalization and ICU admission among immigrants with cancer or MH&A compared to non-immigrants without these conditions. A critical theme that emerged from the lived experiences or clinical observation of panelist discussion was the persistent shortage of primary care providers, which panelists identified as a significant issue disproportionately impacting immigrant populations, particularly during the COVID-19 pandemic. While this challenge predates the pandemic, it was notably intensified by the crisis. The widespread closure of healthcare facilities and offices further limited access to essential services, posing additional barriers for individuals with limited English proficiency and making it even harder to seek care or support. Panelists also reflected on the data revealing the economic, social, and structural disadvantages faced by immigrants during the pandemic. They emphasized that, for many immigrant communities, the daily struggle to meet basic needs often overshadowed health priorities, such as getting vaccinated or accessing the limited healthcare services provided at the time.

The MH&A disorders panel discussion similarly featured two members of the research team (M.V. and A.L.) who presented the study findings, along with a patient/caregiver and two healthcare providers/policymakers. Together, they engaged in a comprehensive discussion of the administrative health data. As with the cancer panel, limited access to primary care providers emerged based on lived experienced and clinical observation of panelist as a central theme. The panelists discussed how this issue, combined with the compounded impacts of COVID-19 on immigrants living with MH&A disorders, resulted in significant downstream effects. These included reduced vaccination rates, the spread of misinformation, and substantial barriers to accessing essential services, testing, and timely diagnoses. Furthermore, the panel reflected on how the pandemic exacerbated pre-existing disparities in mental healthcare access, particularly for immigrant populations facing language, cultural, and systemic challenges. These compounded barriers hindered not only the physical health but also the mental well-being of these communities. The discussion also touched on the need for more integrated, culturally competent healthcare services and improved communication strategies to combat misinformation and ensure equitable access to mental health support, particularly during crises.

### 3.2. Small Group Discussions—Cancer

The 40 Cancer Think Tank participants were divided into four smaller groups of eight to eleven participants: one group of policy and decision-makers, one of healthcare providers, one of patients, and one with a mix of all participant types. During their discussion participants reflected on their personal experiences, observations and suggestions for the reasons immigrants experienced more compounded disadvantages during COVID-19. Nine key themes emerged:

Lack of primary care physicians: Immigrants faced significant barriers in accessing timely primary care.

Exclusion of foreign-trained healthcare providers: The inability of foreign-trained healthcare providers to integrate into the healthcare system was identified as a missed opportunity to alleviate workforce shortages.

Need for more culturally sensitive system navigation and outreach: Participants highlighted the importance of tailored support for navigating healthcare services.

Poor communications during COVID-19: Insufficient or unclear public health messaging disproportionately affected immigrant communities.

Inequitable access to healthcare: COVID-19 further widened existing disparities in access to care.

Disconnect between decision-makers, service providers, and communities: Participants emphasized the need for stronger collaboration and alignment of messaging between these groups.

Need to Amplify community voices: Including immigrant communities in decision-making process was strongly highlighted.

Compounded language barriers: Limited language support during the pandemic created additional challenges.

Disruption of prevention programs: Preventive care services were halted, leaving many without essential screenings and early interventions.

#### 3.2.1. Personal Experiences Shared by Participants

Participants shared firsthand accounts illustrating the challenges faced by immigrants living with cancer during COVID-19. One patient participant described their difficulties navigating the healthcare system as a new immigrant:

“[I was] vaccinated 114 days before entering Canada as [a] new immigrant with cancer. Did not have OHIP. Did not find [a] family doctor. Did not know how to access care. Could not receive follow-up COVID-19 vaccination”.

Another participant highlighted the challenges of housing and caregiving while undergoing treatment:

“[I] had difficulty isolating in a one-bedroom apartment with me taking chemo and my son having COVID. I was not sure how to manage, and no one was available to respond to my questions”.

Participants also discussed strategies they employed to access care. One patient described relying on informal networks:

“Family and friends told us to go to walk-in clinics and through them be referred to cancer-related healthcare”.

#### 3.2.2. Challenges Faced by Service Providers

Service providers emphasized systemic barriers and service limitations that hindered access to care for immigrants. One noted the restrictive availability of services:

“Most services are only open during the weekdays. If the patient is working or is in and out of hospitals during the weekday, then they are deprived of services”.

These discussions underscored the multifaceted barriers immigrants faced during COVID-19 and the urgent need for systemic changes to ensure equitable access to cancer care.

#### 3.2.3. Small Group Discussions—MH&A Disorders

The 41 MH&A Disorders Think Tank participants were divided into four groups of seven to eleven participants: one group of decision-makers and service providers, one group of service providers, one group of patients, and two mixed groups of all participant types. From their discussions, the following five major themes emerged, providing key insights into the challenges and experiences of individuals with MH&A disorders during COVID-19.

**Lack of Access to Services and Care**

Participants highlighted significant barriers to accessing care, exacerbated by the closure of key services, lack of information, and inadequate navigation support. An affected community member living with MH&A shared the following:

“There is a lack of psychiatrists, there is a lack of counselors. And then again I find that geographical boundaries, like if I’m in Mississauga, I cannot go to Milton if I see a resource over there, or I cannot go to downtown Toronto”.

The pandemic intensified these challenges. For example, a community member recounted the following:

“I wonder how many other people who were in the middle of diagnosis suffered getting treatment. I heard from one individual that there was only virtual diagnosis. They could see how the child interacted, wrote, or colored, but the assessment was questionable to the parents regarding the severity of autism spectrum disorder or other special needs”.

The worsening of MH&A conditions during COVID-19 created vulnerable situations. A caregiver for an immigrant living with MH&A shared their experience:

“Managing my son, diagnosed with mental health issues since 2005, was not easy. During the pandemic, his condition got worse. He threw out his medications and threatened me at knife point, saying, “call the cops”. It took the cops 1.5 h to arrive, and when they did, they broke down our door with force and came with guns. I still cannot forget that scene”.

**Increased Siloing of Systems**

The pandemic further fragmented healthcare and social service systems, leaving many communities struggling to meet their basic needs. A participant emphasized the importance of addressing social determinants of health:

“Before we even engage in those health conversations, people need housing, necessary clothing, and social support like childcare or caregiving to participate in addressing their health needs”.

Participants also noted the division created by the pandemic:

“What we’ve observed with the pandemic were such huge divisions around who’s right, who’s wrong. This further marginalized and isolated some communities, creating additional barriers for people to ask questions and make informed decisions”.

**Different Impacts on Family Members**

The uncertainty, isolation, and public health measures during the pandemic significantly impacted children and youth, with increased anxiety and obsessive-compulsive behaviors fueled by negative media exposure. One participant explained the following:

“That daily reporting of COVID-19 cases scared us more. “Today’s number is this, tomorrow it’s higher”. It increased anxiety. We were in isolation with no clear guidance on how to reduce our stress”.

Another participant (a caregiver) compared lockdowns to imprisonment:

“The lockdown felt like being imprisoned. It was a new life we weren’t accustomed to, and it loaded us with stress, leading to anxiety and depression”.

The mental health of children and youth was a particular concern. One participant shared the following:

“Another depression is growing among teenagers. My kids now spend more time playing alone on computers and don’t want to do anything else”.

The shift to virtual healthcare also created challenges, as one participant explained:

“Youth suffered because they’re not used to interacting with doctors over the phone. They had no one to talk to except friends, if that was even available. The lack of trust or knowledge about mental health and addiction among peers made things worse”.

The pandemic’s isolation was compounded by overcrowded living conditions, which heightened stress and increased family violence. A community member reflected the following:

“The pandemic isolated seniors and women. Living in an overcrowded apartment during lockdown made everyone stressed because they didn’t have free time or their own space often escalating tensions and leading to quarrels and conflicts”.

#### 3.2.4. Complexities of Going Virtual

The transition to virtual services created significant obstacles, especially for those unfamiliar with the technology. As one participant described,

“Appointments became frustrating. You’d wait all day for a quick call, and if you went to the washroom and missed the call, it would be dropped. That alone created anxiety: What now? When will I get another appointment?”

### 3.3. Proposed Action Items

Participants formulated a list of action items, at each Think Tank, and voted on them based on how important and how easy it is to address them. In the Cancer Think Tank, participants identified the following as the most important action items.

#### 3.3.1. Leverage Foreign-Trained Healthcare Providers (HCPs)

Utilize the available workforce of foreign-trained HCPs, particularly within immigrant communities. These individuals can serve as clinical health ambassadors, providing culturally sensitive health information and improving access to services.

#### 3.3.2. Create Clinical Health Ambassadors

Train community-based health ambassadors, particularly from the pool of internationally trained health professionals, to facilitate communication, promote preventive care, and increase awareness of cancer screening services.

#### 3.3.3. Connect Immigrants with Healthcare Providers at Entry

Upon arrival in Canada, ensure that immigrants are connected with healthcare providers and social services. This should begin at the Immigration Health Check, assisting newcomers with navigating the healthcare system and accessing essential health services early on. 

#### 3.3.4. Leverage the Existing Workforce of Foreign-Trained HCPs

With many foreign-trained professionals already in the country, this initiative can be easily implemented by creating pathways for them to serve their communities, reducing the need for extensive additional resources.

#### 3.3.5. Listen to the Community and Engage in Decision-Making

Creating forums where community members are directly involved in decision-making processes and have direct access to policymakers can be relatively straightforward and can help bridge the gap between service providers and recipients. 

In the MH&A disorders Think Tank, participants identified the following actions items as the three most important.

#### 3.3.6. Promote Intersectoral Collaboration

Break down the silos between health, social services, immigration, housing, and finance policies. Integration of these sectors is essential for delivering comprehensive care, especially for immigrants who often face multiple, interconnected challenges.

#### 3.3.7. Empower Communities and Build Capacity

Engage and empower grassroots organizations, cultural groups, and community leaders. By asking communities what they need and providing them with the necessary resources, including capacity building, these groups can lead the charge in mental health and addiction services.

#### 3.3.8. Move Away from a Cookie-Cutter Approach and Instead Embrace Inclusive and Diverse Approaches

Tailor mental health and addiction programs to the specific needs of different communities. This involves actively consulting with communities and adapting services to their unique cultural, social, and economic contexts. Programs must be co-designed to ensure relevance and effectiveness. 

The following ideas were identified as easiest to implement.

#### 3.3.9. Address Computer Literacy for New Immigrants and Seniors

Providing training to new immigrants and seniors in computer literacy will help them navigate online health services and other systems, improving access to mental health and addiction resources. This is a straightforward initiative that can be implemented through community organizations or libraries.

#### 3.3.10. Move Away from a Cookie-Cutter Approach

Asking communities what they need and adapting programs to fit their needs can begin immediately by holding consultation sessions and focus groups. This approach fosters trust and ensures services are relevant and effective.

#### 3.3.11. Provide Cultural Sensitivity and Safety Training

Training administrative staff, gatekeepers, and service providers in cultural sensitivity, competence, and safety is an easy first step to improve the accessibility and inclusivity of mental health and addiction services. This training should be mandatory to ensure equitable service delivery.

## 4. Discussion and Recommendations

Overall, our Think Tanks successfully engaged a broad range of stakeholders to deepen our understanding of the compounded impacts of COVID-19 on immigrants living with cancer and/or mental health and addiction (MH&A) disorders. By convening a panel of experts to whom we presented our critical analysis of provincial administrative data, we were able to foster important discussions on effective and inclusive strategies that aim to reduce health disparities and promote equitable access to cancer and MH&A services during pandemics or other future crises. Drawing on participants’ brainstormed ideas and their assessment of the importance and feasibility of various solutions, we developed a set of actionable recommendations for addressing the compounded impacts of pandemics and crises on immigrants living with chronic conditions.

Based on the findings from the Cancer Think Tank, we recommend four key action items that directly stem from the outputs of the Think Tank, to address the unique challenges faced by immigrants living with cancer during pandemics and future health crises. First, leveraging foreign-trained HCPs as community health ambassadors can serve as a crucial strategy. Foreign-trained HCPs as trusted members of immigrant communities, can offer culturally relevant health information, promote cancer prevention and screening, and assist individuals in navigating the healthcare system. Their involvement can build trust and improve health outcomes for underserved populations. Second, establishing connections with health and social services at the Immigration Health Check can provide immediate support to immigrants and refugees as they arrive in Canada. By ensuring they are connected to healthcare providers and social services from the outset, this early intervention can help immigrants navigate the healthcare system more efficiently and ensure timely access to care. Third, expanding access to community health centers and cancer screening services to enable health connections for all is essential. Increasing the availability of community health centers in underserved areas and improving access to cancer screening services will reduce wait times, promote preventive care, and facilitate earlier cancer detection among immigrant populations. Lastly, creating a provincial health portal with culturally responsive information is another critical recommendation. An online resource offering simple, culturally safe, and easily understandable health information would enable immigrants and refugees to access important health-related resources in their own language, improving communication, accessibility, and health literacy.

Drawing directly from the discussions and voting in the MH&A disorders Think Tank, we recommend four action items to address the compounded impacts of pandemics and future crises on immigrants living with MH&A disorders. First, promoting intersectoral collaboration across services is crucial. Integrating health, social services, immigration, housing, and finance policies ensures that care for immigrants facing MH&A disorders is comprehensive and holistic. By breaking down silos between sectors, we can offer more effective support to address the interconnected needs of individuals. Second, empowering communities and tailoring programs to local needs is essential. Engaging communities in decision-making processes, asking them about their needs, and adapting services to fit their cultural and social contexts fosters trust and ensures that mental health and addiction programs are relevant, effective, and culturally responsive. Third, enhancing access to mental health services through cultural competency training is necessary to ensure that frontline staff and service providers are equipped to offer inclusive care. Providing mandatory cultural sensitivity, competence, and safety training will bridge gaps in understanding and improve the quality of mental health and addiction care provided to immigrant and marginalized populations. Lastly, increasing digital and health literacy for newcomers and vulnerable groups is critical. Programs aimed at improving computer literacy and health navigation skills, particularly for new immigrants and seniors, will help these populations access mental health resources more effectively and navigate the Canadian healthcare system.

These recommendations are designed to foster a more inclusive and responsive healthcare system, addressing the unique needs of immigrants living with chronic conditions and ensuring that future crises do not exacerbate existing health disparities.

## 5. Limitations

The results of our findings should be examined while considering a few limitations. While efforts were made to address language barriers by providing interpreters during the Think Tank sessions, some cultural or linguistic nuances may still have been overlooked like indirect language or metaphor that was intended to convey patient’s concern or disagreement was translated verbatim altering the intended message or tone. Additionally, the Think Tanks may not have included all relevant stakeholder groups, such as certain immigrant subpopulations or younger individuals, potentially limiting the diversity of perspectives.

There may also have been some selection bias, as participants who attended were likely already engaged or vocal about the issues discussed, possibly excluding harder-to-reach or less vocal communities, such as newly arrived immigrants or those without access to technology. The time constraints of the sessions further restricted the depth of exploration into complex issues, potentially leading to an oversimplification of nuanced challenges and solutions in certain instances. Lastly, the insights were primarily drawn from participants’ personal experiences and observations, which may lack empirical validation, making it harder to generalize findings to broader populations.

## 6. Conclusions

The Think Tanks provided a valuable platform to hear directly from affected communities about how COVID-19 impacted immigrants living with cancer and/or mental health and addiction (MH&A) disorders. These sessions allowed us to move beyond administrative data to gain a deeper understanding of personal experiences, while facilitating dialog between community members, policymakers, decision-makers, and healthcare providers.

By fostering collaboration at the community level, we critically discussed and developed recommendations to address the impacts of COVID-19 and prepare for future crises affecting immigrants with chronic health conditions. Our findings highlight that immigrants living with cancer and/or MH&A disorders face heightened vulnerabilities, which are further exacerbated during pandemics.

Through these discussions, we identified key intervention priorities, including the following: recognizing the role of culture in care delivery; expanding access to healthcare; targeting critical points along immigration and settlement pathways; promoting intersectoral collaboration for a holistic approach to care; and enhancing health literacy and competency among both service users and providers.

## Figures and Tables

**Table 1:** Questions asked during Think Tank.

Questions asked during panel discussion of the results
What do you find most surprising about the data? What about these findings inspires you to take action and/or speak to others about taking action? What makes you hopeful that positive change can happen and what that change might look like?
Questions asked during small group discussions
How do your experiences either differ from or support the research findings? (secondary prompting question if needed—what is your understanding/experience of health inequities of immigrants during COVID-19?) What needs to be done to reduce current and future health pandemic disparities? (secondary prompting question if needed—what can be done by individuals, families, communities and at the system level? What is the #1 action or change that you think needs to happen next?” (secondary prompting question if needed—if we want to decrease health disparities now and in the future what do we need to do first?)

**Table 2:** Think Tank Participants.

Cancer Think Tank Participants	
Total Number of Registrants	39
Total number of session participants	40
Number of patient/care giver participants	10
Number of healthcare provider participants	12
Number of decision-maker/policy participants	12
Number of research team and collaborator participants	6
Mental Health and Addictions Disorders Think Tank Participants	
Total number of registrants	47
Total number of session participants	41
Number of patient/care giver participants	15
Number of healthcare provider participants	12
Number of decision-maker/policy participants	8
Number of research team and collaborator participants	6

## Data Availability

The datasets generated and/or analyzed during the current study are not publicly available due to maintaining the privacy and confidentiality of participants, but are available from the corresponding author on reasonable request.
